# Drug Related Hospital Admissions; A Systematic Review of the Recent Literatures

**DOI:** 10.29252/beat-070401

**Published:** 2019-10

**Authors:** Mohammed Biset Ayalew, Henok Getachew Tegegn, Ousman Abubeker Abdela

**Affiliations:** 1 *Department of Clinical Pharmacy, School of Pharmacy, College of Medicine and Health Sciences, Gondar University, Gondar, Ethiopia*

**Keywords:** Drug related problems, Hospital admission, Adverse drug reaction, Review, Emergency visit.

## Abstract

**Objective:**

To derive findings from different studies done on drug related hospital admissions and comprehensively express the incidence and preventability of drug related hospital admissions; identify the common types of drug related problems that caused hospital admission, and identify factors associated with drug related hospital admission.

**Methods:**

Literatures that assessed hospitalization due to drug related problems were searched online using Pub Med and Google Scholar databases. The relevant reference lists of retrieved articles were also searched manually on Google. Prospective and retrospective studies conducted anywhere in the world on drug related hospitalization, published from January 2012 to January 2017 as an original article and written in English language were included.

**Result:**

The prevalence of drug related hospital admission varies from 1.3% to 41.3% with the average rate of 15.4%. Among hospitalized patients 2.7% were died due to drug-related problems (DRPs). Drugs that were frequently reported as causing drug related admission were antithrombotic drugs, antihypertensive drugs, analgesics, anti-diabetics, antipsychotics, and anti-neoplastic drugs. Poly pharmacy, old age and female sex were mentioned as determinants for drug related hospitalization by a number of studies. About one third of drug related hospital admissions were definitely preventable and more than 40% were also potentially preventable.

**Conclusion:**

Drug related problems contribute for more than 15% of hospital admissions. Higher risk of admission due to DRPs was observed in patients who were on poly pharmacy and those who were old. As most of drug related hospital admissions were preventable an emphasis should be given for preventive strategies to avoid complications and costs associated with admission.

## Introduction

World-wide, medication use is increasing. This can be explained as a result of production of more types of medications by the advancing pharmaceutical industries and the ever increasing types of diseases that amplified needs of pharmaceuticals. Possible outcomes of medication use may range from the intended beneﬁcial effect interventions to minor side effects and even death. Drug related problem (DRP) is deﬁned as an event or circumstance that involves a patient’s drug treatment that actually, or potentially, interferes with the achievement of an optimal outcome [1, 2]. This can be inappropriate drug selection, adverse drug reactions, untreated indication, drug interactions, inappropriate dosage, drug use without indication and non-compliance.

Admissions due to DRPs have been reported as growing over the past decades [3]. In United States, estimates suggested that Drug-related problems (DRPs) accounted for 17 million emergency department visits and 8.7 million hospital admissions annually [4]. Evaluation of studies on DRPs which leads to hospital admission have indicated that DRPs are responsible for approximately 5-15% of all hospitalizations, of which 25-75% were avoidable [5-11]. Those medications with narrow therapeutic index and medications which require continuous and regular monitoring are the one most implicated in avoidable adverse drug events (ADEs) [5]. A number of factors have been implicated to be risk factors for drug related hospital admission. Examples include being old [3, 7, 12, 13], Poly pharmacy [3, 14], poly physician [14], being female [15], and presence of co morbidities [5, 16, 17]. Drug related hospitalization will have negative consequences on patients and society. It increases mortality and morbidity rates, health care cost, decreases income and household productivity and reduced quality of life [5, 18].

Different studies across the world reported varying level of incidence and preventability of drug related hospital admission based on the settings, methods used and populations addressed. It is very important to see the current overall picture of drug related hospitalization to understand the nature and extent of the problem and to devise strategies for preventing its harm. So the aim of this review is to derive findings from different studies done on drug related hospital admissions and comprehensively expresses the incidence and preventability of drug related hospital admissions; identify the common types of DRPs that caused hospital admission, and identify factors responsible for drug related hospital admission.

## Materials and Methods

*Search strategy*

Literatures that assessed admission to an emergency department or other units of the hospital due to adverse drug reactions or any other drug related problems were searched online using Pub-med and Google Scholar databases. The following words were used in different combinations during online search; Drug related problem(s), hospitalization, emergency department visit, adverse drug reaction, hospital admission and drug related admission. The relevant reference lists of retrieved articles were also searched manually on Google.

*Article selection*

All of the identified articles were independently reviewed by three authors to assess eligibility for inclusion in the review. Disagreements were resolved by consensus. Prospective and retrospective studies conducted anywherein the world on drug related hospitalization, published in January 2012 to January 2017 as an original article and written in English language were included in the study. Studies conducted in the pediatric population were excluded. 

*Data extraction*

Information regarding study characteristics (study area, study design, subjects and sample size) and main findings (frequency of hospital admission and death due to drug related problems, type, severity, casualty and preventability of DRPs, drugs and drug classes responsible for admission and factors associated with drug related admission) were extracted from each studies.

## Results

*Literature search results*

**Fig. 1:**
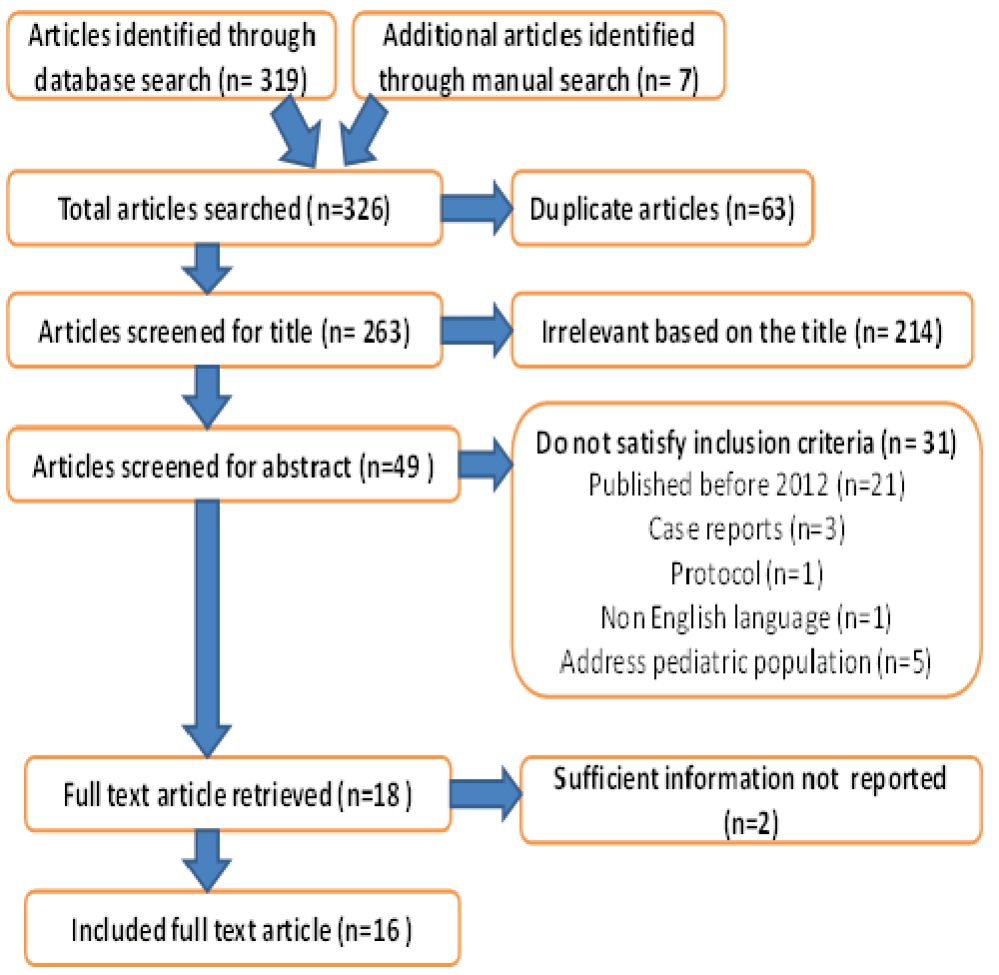
Flow diagram of study selection

A total of 326 articles were obtained from Pub med, Google Scholar and manual Google search. After adjusting for duplicates 263 articles remained. Of these 214 studies were found irrelevant after reviewing their titles. Thirty-one studies were discarded as they do not satisfy the inclusion criteria after reviewing their abstracts. The full text of the remaining 18 studies was reviewed in detail. Two studies were removed after the full text was reviewed since it did not address many of the needed information. Finally, as shown in Figure 1, sixteen studies were found convenient to be included in this review. 

*Study characteristics*

Among the 16 studies reviewed majority (12) assessed admission to any department of the hospital while 4 assessed admissions to emergency department only. Nine of the studies reported hospitalization due to any type of drug related problems and the other 7 assessed admissions due to adverse drug reactions only. Nine of the studies included all adult patients while others include only specific patient groups like cancer patients (1), cardiac transplanted patients (1), elderly patients with dementia (1), patient who are age 65 or more (2), age 60 or more (1) and age 55 or more (1). As shown in Table 1, the studies were conducted in different parts of the world on samples of 48 –2,127,133 patients. Most (11) of the studies were prospective in design.

*Drug related hospitalization and death*

There is no significant difference in the rate of drug related hospitalization between the large and small sample size studies (*p*=0.268). As indicated in Table 2, the rate of drug related hospital admission varies from 1.3% in Netherlands to 41.3% in Sweden. The average rate of drug related hospital admission is 15.4%. Seven studies reported that 0% up to 5.7% of patients hospitalized due to drug related problems were died. The average death rate due to DRPs in hospitalized patients is 2.7 %. 

**Table 1:** Individual study characteristics

**Sr. no.**	**Author, year of publication**	**Study area/country **	**Study subjects**	**Study design**	**Sample size**
**1**	Alghamdy M. et al, 2015 [20]	Saudi Arabia	Admitted patients at emergency department	Retrospective record review	5622
**2**	Chan A. et al, 2014 [21]	Singapore General Hospital	cancer patients admitted to two oncology wards	prospective, observational study	1274
**3**	Schmiedl, S et al, 2014 [22]	German	patients admitted to the internal medicine departments	multi-centre, Prospective study	212,000
**4**	Benard-laribiere A. etal, 2015 [23]	61 Medical wards in public hospitals of France	Patients admitted to the medical wards	Prospective study	2692
**5**	ReppK.. et al, 2012 [5]	Saint Luke's North Hospital, Kansas City, USA	cardiac transplant patients	prospective longitudinal - single center study	48
**6**	Al-Arifi M. et al, 2014 [4]	Saudi Arabia	Patients visiting emergency department	prospective cohort observational study	300
**7**	Nickel C. et al, 2013 [26]	University Hospital Basel , Switzerland	non-trauma patients presenting to the ED with non-specific complaints	Prospective crossectional study	633
**8**	Gustafsson M. et al, 2016[16]	Sweden	Elderly patients with dementia or cognitive impairment admitted orthopedic and internal medicine wards	NR	458
**9**	Jatau A. et al, 2015 [18]	UniversitiSains Hospital, Malaysia	patients who visited the ED	Prospective cross-sectional study	434
**10**	Ruiter R*et *al, 2012 [10]	Hospitals in Netherlands	patients >55 years of age with an acute, non-planned admission to a Dutch hospital	Retrospective study	2, 127,133
**11**	Pedrós C.et al, 2016 [24]	Bellvitge University hospital, Barcelona, Spain.	Patients aged >65 years who were urgently admitted at the hospital	prospective cross-sectional study	60,263
**12**	Ahern F, et al, 2013 [32]	Cork University Hospital (CUH) emergency department, Ireland	Patients admitted to the ED	retrospective study	856
**13**	Pedrós C. et al, 2014 [25]	Bellvitge University Hospital, Spain	patients admitted through the emergency room	prospective cross-sectional study	4,403
**14**	Karuppannan M. et al, 2013 [27]	Two medical wards at a government hospital in Malaysia.	Patients admitted to medical wards	Prospective study	1200
**15**	Skoldunger A. et al, 2015 [34]	four sites in Sweden	individuals aged ≥60 years	Longitudinal prospective cohort study	4108
**16**	Marcum Z. et al, 2012 [33]	Veterans Affairs (VA) Medical Centers, USA	older (aged ≥65)	Retrospective cohort	678

^a^USA; United States of America

**Table 2:** Rate of drug related hospital admission and death and types of DRPs causing hospitalization

**Sr. no.**	**Author, year of publication**	**Admission to Hospital (reason)**	**Death due to DRPs**	**Types of DRPs that causes admission**
**1**	Alghamdy M. et al, 2015 [20]	253 (4.5%) (due to DRP)	10 (4%)	Noncompliance 112 (44.3%) Toxicity and SEs 50 (19.8%) Drug-drug interactions 29 (11.5%) Accidental and suicidal drug ingestions 26 (10.3%) Drug abuse 18 (7.1%) Drug allergy 10 (4%) Super-infections 8 (3.2%)
**2**	Chan A. et al, 2014 [21]	158 (12.4 %) (due to DRP)	5 (3%)	Adverse reaction 155 (94.5%) Drug interactions 3 (1.8%) Dosing problem 3 (1.8%) Drug use problem 3 (1.8%)
**3**	Schmiedl, S et al, 2014 [22]	6887(3.2%) (due to ADR)	1.32%	NR
**4**	Benard-laribiere A. et al, 2015 [23]	97(3.6%) (due to ADR)	1%	Type A reactions 67 (69.1%) Vascular disorders 20 (20.6%) CNS disorders 11 (11.3%) Gastrointestinal disorders 9 (9.3%) General disorders 9 (9.3%).
**5**	ReppK. et al, 2012 [5]	40% (19/48) (due to DRP)	0%	Adverse drug reactions 6 (32%) Supra-therapeutic doses 6 (32%) Sub-therapeutic doses 3 (16%) Untreated indication 1 (5%) Non-adherence 2 (11%) Drug interactions 1 (5%)
**6**	Al-Arifi M. et al, 2014 [4]	56 (18.7%) (due to DRPs)	NR	Adverse drug reactions (30.4%) non-compliance (30.4%) Untreated indication (10.7%) Drug interactions (7.1%) Supra-therapeutic (7.1%) Sub-therapeutic dose (7.1%) Improper drug selection (5.4%) drug use without indication (1.8%)
**7**	Nickel C. et al, 2013 [26]	77 (12.2%) (due to DRPs)	NR	Adverse drug reactions 42 (56%) Drug choice problem 9 (12%) Dosing problems 16 (21%) Drug use problems 2 (3%) Drug interactions 3 (4%)
**8**	Gustafsson M. et al, 2016 [16]	189 (41.3 %) (due to DRPs)	NR	Dosage too high (12.7 %) Noncompliance (10.6 %) Ineffective drug 10.6 % interactions 6.9 % Needs additional drug (6.3%) Dosage too low (4.8 %) Unnecessary drug therapy (2.6 %)
**9**	Jatau A. et al, 2015 [18]	133 (30.6 %) (due to ADE)	NR	Therapeutic failure74 (55.6 %) ADR 43 (32.3 %) Accidental overdose 7 (5.2 %) Intentional overdose 6 (4.5 %) Untreated indication 3 (3.2 %)
**10**	Ruiter Ret al, 2012 [10]	26852 (1.3%) (Due to ADR)		ADR
**11**	Pedrós C.et al, 2016 [24]	1976 (3.3%) (Due to ADR)	113 (5.7 %)	Acute renal failure (22.9 %) Upper GI bleeding (16.6%) Lower GI bleeding (11.2 %) Intracranial bleeding (9.3%) Digitalis intoxication (3.7 %)
**12**	Ahern F, et al, 2013 [32]	75 (8.8%) (due to ADR)	NR	NR
**13**	Pedrós C. et al, 2014 [25]	4.2 % (due to ADR)	3.2 %	Type A reactions 171 (91.9 %) Type B 15 (8.1 %) Renal & urinary disorder 59 (29.4 %) Gastrointestinal disorders 53 (26.4 %) Nervous system disorders 21 (10.4 %)
**14**	Karuppannan M. et al, 2013 [27]	443 (39%) (ADR)	NR	Treatment Failure 351 (79%) ADR 94 (21%) Drug overdose 21 (5%) Medication error 15 (3%)
**15**	Skoldunger A. et al, 2015 [34]	536 (13%)	NR	NR
**16**	Marcum Z. et al, 2012 [33]	68 (10%) (due to ADR)	NR	Bradycardia (n = 6) hypoglycemia (n = 6) falls (n = 6) mental status changes (n = 6)

**Table 3:** drugs and other factors that are associated with drug related hospitalization

**Author, year of publication**	**Drugs that cause DRP**	**Factors significantly associated with admission due to DRPs**
Alghamdy M. et al, 2015 [20]	Antiepileptic drugs Paracetamol Opioid Benzodiazepine Antibiotics Antisecratory drugs Antipsychotic drugs Antihypertensive drugs	NR
Chan A. et al, 2014 [21]	Cyclophosphamide (n=35), Doxorubicin (n=25), Cisplatin (n=25), Docetaxel (n=18), Paclitaxel (n=16).	Young age (p=0.03) Female sex (P=0.015)
Schmiedl, S et al, 2014[22]	Antithrombotic agents drugs used in diabetes NSAIDs Paracetamol	NR
Benard-laribiere A. etal, 2015 [23]	Antithrombotic 12.6% Antineoplastic agents 12.6% Diuretics 9.0% Analgesics 9.0% Anxiolytics/hypnotics/antipsychotics 6.6%	Old age (P<0.001)
ReppK. et al, 2012 [5]	Immunosuppressant (63%) Antimicroblal (11%) Electrolyte/Fuid (11%) Anticoagulant (5%)	NR
Al-Arifi M. et al, 2014 [4]	Antihypertensive agents (21.5%) Anticoagulants (14.3%)Immunosuppressant (12.5%) Chemotherapeutic agents (10.7%)	NR
Nickel C. et al, 2013 [26]	Thiazides Benzodiazepines Antidepressants Anticonvulsants	NR
Gustafsson M. et al, 2016 [16]	Cardiovascular drugs (29.5 %) Psychotropic drugs (27.3 %)	Poly-pharmacy
Jatau A. et al, 2015 [18]	Antidiabetics (23.3 %) Antihypertensives (21.1 %) Antibiotics (9.8 %) Anti-asthmatics (8.3 %) Diuretics (6.0 %)	NR
Ruiter Ret al, 2012 [10]	Anticoagulants Antineoplastic and immunosuppressive drugs Antidiabetic agents High-ceiling diuretics Salicylates Antirheumatics	Age >75 years Female sex
Pedrós C.et al, 2016 [24]	High-ceiling diuretics ACE inhibitors Cardiac glycosides	NR
Ahern F, et al, 2013 [32]	Diuretics (n=22), Aspirin (n=5) Warfarin (n=4)	Poly-pharmacy
Pedrós C. et al, 2014 [25]	Diuretics 69 (18.1 %) Antithrombotic drugs 63 (16.5 %) RAAS inhibitors 56 (14.7 %) NSAIDs 43 (11.3 %)	Age >65 years Poly-pharmacy
Karuppannan M. et al, 2013 [27]	Antidiabetic 36 (38.3%) Antiplatelet 10 (10.6%) Thiazide diuretic 10 (10.6%) ACE Inhibitor 10 (10.6%) CCB 10 (10.6%)	NR
Skoldunger A. et al, 2015 [34]	NR	Old age, Male sex, Living at home (community dwelling), Lower educational level, Functional dependence, Multiple co-morbidity.
Marcum Z. et al, 2012 [33]	Beta-blocker Oral hypoglycemic agent Thiazide diuretic Anticoagulant Antidepressant NSAIDs	Poly-pharmacy

**Table 4:** Preventability of DRPs that caused hospital admission

**Author, year**	**Not preventable (%)**	**Potentially preventable (%)**	**Definitely preventable (%)**
Alghamdy M. et al, 2015 [20]	NR	70	NR
Chan A. et al, 2014 [21]	45.7	52.4	15.2
Benard-laribiere A. et al, 2015 [23]	16.5	16.5	32
ReppK. et al, 2012 [5]	NR	NR	58
Al-Arifi M. et al, 2014 [4]	14.3	53.6	32.1
Jatau A. et al, 2015 [18]	33.1	11.3	55.5
Ahern F, et al, 2013 [32]	33.3	52	5.3
Marcum Z. et al, 2012 [33]	NR	NR	36.8
Average	28.6%	42.6%	33.6%

*Drug related problems that cause hospital admission*

As shown in Table 3 the common drug related problems that caused hospital admission were adverse drug reaction, dosing problems, noncompliance, untreated indication and improper drug selection. 

*Drugs and other factors that are associated with drug related hospitalization*

Drugs that were frequently reported as causing drug related admission were antithrombotic drugs, antihypertensive drugs, analgesics, anti-diabetics, antipsychotics, and anti-neoplastic drugs. Poly pharmacy, old age and female sex are mentioned as factors for drug related hospitalization by a number of studies. Presence of multiple co morbidity, lower educational level, functional dependence, young age and male sex were also mentioned in some studies as a factor that influence hospital admission due to drug related problems.

*Preventability of DRPs that caused hospital admission*

Around one third of drug related hospital admissions were definitely preventable and more than 40 % were potentially preventable. Report of the reviewed studies on the Preventability of Drug related hospitalization is indicated in Table 4.

## Discussion

This systematic review summarizes prevalence of hospital admissions due to drug-related problems and preventability of drug related hospital admissions. In addition, the study has also extorted findings to identify the common types of DRPs that caused hospital admission, and associated factors responsible for drug related hospital admission from 16 different studies published recently.

The rate of drug related hospital admission varies from 1.3% to 41.3% with the average rate of 15.4%. This figure indicated that the burden of DRPs as a cause of hospitalization is very significant. In addition to hospital admission, DRP may contribute to poor patients’ clinical outcome at discharge such as disability or death. Chiefly ADRs can be potentially lethal and are a main cause of mortality [19]. The mortality rate among patients hospitalized due to drug related problems as reported in the seven studies included in this review [5, 20-25] was up to 5.7% with the average death rate of 2.7 %. 

The types of DRPs causing hospital admission varied across the studies reviewed. Among the twelve studies that have reported types of DRPs causing hospital admission, six [4, 5, 16, 20, 21, 26] have explicitly reported the frequencies of each type of DRPs responsible for hospital admission. As a result, ADR in 4 studies [4, 5, 21, 26], noncompliance in Alghamdy*et al*., [20]study and dosage too high in Gustafsson*et al*. [16] study were identified as the most frequently occurring types of DRPs that caused hospital admission. However, Jatau*et al*., [18] and Karuppannan*et al*., [27] have assessed prevalence of admissions due to ADE and both studies identified treatment failure as the most frequent cause of admission, which is in contrast to previous studies published before 2012 where ADRs was most common type of ADE accounted for 53-90% of ADE related admissions [1, 28-30] followed by therapeutic failure [31]. The other four studies [10, 23-25] in this review have determined ADR specific hospital admission and hence, Type A reaction in two studies [23, 25] and acute renal failure in Pedrós*et al*.,[24] study have been reported to be the most common subtypes of ADR that caused hospital admission. 

There have not been any standardized methods of classifying drugs making it difficult for comparisons as to which drug causes drug related hospital admission across different studies. Concordant with the studies included in the present review [10, 16, 18, 24, 25, 32] a previous review that took studies published before 2012 mentions, cardiovascular drugs (antithrombotic drugs, antihypertensive drug), antineoplastic agents, anti-inflammatory and antidiabetic agents as a commonly reported drug classes causing hospital admission [3].

Age is the most frequently mentioned factor for drug related hospital admission. In line with the result of this review old age was identified as a major risk factor for drug related hospital admission in many other studies [3, 7, 12, 13, 17]. The main reason for geriatric population to be more susceptible for admission due to DRPs could be due to physiological and pathological changes occurred at advanced age which leads to a change in pharmacodynamics and pharmacokinetics of a drug in elderly. Drug absorption, distribution metabolism and excretion will be significantly affected in older people which may subsequently lead to toxicity and complication which necessitates hospitalization. In addition polypharmacy [16, 25, 32, 33], gender [10, 21], presence of multiple co morbidity [34], lower educational level and functional dependence [34] were also mentioned as a factor that influence hospital admission due to drug related problems. Similarly previous studies also agree that the above mentioned factors have significant association with drug related hospital admission [3, 5, 7, 12-17].

According to the current review significant numbers of DRPs were found to be either potentially or definitely preventable. Hence, several strategies can be designed to minimize those preventable DRPs, thereby curbing the risk of hospital admission. Health care providers should be aware of the common risk factors so as to lower hospital admission due to DRPs. Furthermore, prescribers are in a better position to lessen inappropriate prescribing and identifying the associated factors with DRPs. Pharmacists should also be involved in medication review and reconciliation and actively participate in minimizing poly pharmacy so as to lower the incidence of drug related admissions. 

Drug related problems contribute for more than 15% of hospital admissions. Higher risk of admission due to DRPs was observed in patients who were on poly pharmacy and those who were old. As most of drug related hospital admissions were preventable an emphasis should be given for preventive strategies to avoid costs and complications associated with admission.
